# Incidence and Associated Factors of Weight Gain During the Covid-19 Pandemic

**DOI:** 10.3389/fnut.2022.818632

**Published:** 2022-02-24

**Authors:** Marina Martins Daniel, Juliana Costa Liboredo, Lucilene Rezende Anastácio, Tamires Cássia de Melo Souza, Lívya Alves Oliveira, Ceres Mattos Della Lucia, Lívia Garcia Ferreira

**Affiliations:** ^1^Graduate Program in Nutrition and Health, Department of Nutrition, Universidade Federal de Lavras, Lavras, Brazil; ^2^Department of Food, Universidade Federal de Ouro Preto, Ouro Preto, Brazil; ^3^Graduate Program in Food Science, Universidade Federal de Minas Gerais, Belo Horizonte, Brazil; ^4^Graduate Program in Nutrition Science, Department of Nutrition and Health, Universidade Federal de Viçosa, Viçosa, Brazil

**Keywords:** SARS-CoV-2, coronavirus, social distance, quarantine, obesity

## Abstract

**Introduction and Aims:**

The behavioral changes that arose from quarantine due to the COVID-19 pandemic may have impacted the weight of people. This study aims to investigate the incidence and predictors of weight gain during the quarantine period.

**Methods:**

An online survey was performed five months after the social distance measures implementation. Participants recorded their current and usual weight before lockdown. A multivariate logistic regression model was performed.

**Results:**

Data on 1334 participants were evaluated (33.6y, 79.8% females), and 58.8% have gained weight (3.0 kg; 0.1 to 30.0 kg). Predictors of weight gain were increased food intake (OR = 5.40); snacking (OR = 2.86); fast food (OR = 1.11); canned products (OR = 1.08); and in physical activity (OR = 0.99) concerning the period before the pandemic; also time spent at work, including household chores (OR = 1.58); evening snack (OR = 1.54); higher frequency of alcoholic beverage intake (OR = 1.59) and dose of alcoholic beverage (OR = 1.11); uncontrolled eating (OR = 1.01), and vegetable intake (OR = 0.92) during the quarantine and physical activity before pandemic period (OR = 0.99).

**Conclusion:**

Most participants have gained weight during the pandemic because of working changes, lifestyle, eating habits changes, and uncontrolled eating behavior. These results can be useful to encourage changes during future quarantine periods to prevent weight gain.

## Introduction

The infection of the SARS-CoV-2 virus has already killed over 540.000 people in Brazil (1). Until there is an effective vaccine or treatment, social distancing and self-hygiene are the most effective measures against the spread of the SARS-CoV-2 virus. However, social changes occurring from quarantine impact lifestyle, eating habits (2), and promote sedentary behaviors, such as exposure to screens, teleworking, and the closing of sports spaces and gyms, causing a reduction in energy expenditure (3). These changes in the social environments and changes in the dietary pattern may have contributed to an increase in body weight in the population (4). Some people may adopt an unhealthy diet during the quarantine period, leading to substantial weight gain and, possibly, an increase in the incidence of obesity-related comorbidities (5). Weight gain during quarantine has been described in previous studies (2–7).

Obesity has been pointed out as an important risk factor for aggravation and mortality for COVID-19, especially in younger individuals (8). The impact of obesity on pulmonary function includes decreased expiratory reserve volume, functional capacity, respiratory system compliance (9). Furthermore, obese individuals also have more cardiovascular diseases and type 2 diabetes mellitus, both of which are risk factors for COVID-19 severity and mortality (10).

Based on the latest nationwide surveys carried out during a non-pandemic context, Brazil is already in a situation of high prevalence of obesity (26.8%) and overweight (61.7%) (11). In the current context, changes in the habits of the population during the COVID-19 pandemic (2) are already observable. That way, it is necessary to study the factors associated with weight changes, so that it shall be possible to significantly intervene in the potential future impacts to the health of the population. As far as we know, there are data on how the quarantine period affected eating behavior and weight gain (12), and physical activity (13) among a Brazilian sample. However, there is no more comprehensive data on predictors of weight gain. Thus, the study aimed to investigate the incidence and predictors of weight gain concerning socioeconomic factors, employment status, and social isolation caused by the pandemic, eating behavior, stress, eating habits, and lifestyle during the quarantine period in Brazil.

## Materials and Methods

A cross-sectional online survey created on the Google Form platform® was performed from August 14th to September 9th, 2020., 5 months after the social distance measures implementation. Initially, the researchers shared the link via emails, university websites, and social media (Facebook, Instagram, LinkedIn, and WhatsApp). Participants were also asked to share the link with acquaintances, family, and friends to participate in the research. The entire form took approximately 15 min to complete.

The inclusion criteria were being a resident in Brazil and being 18 years old or older. Pregnant women, incomplete questionnaires, repeated answers were excluded. The study was conducted according to the Declaration of Helsinki. The Research Ethics Committee approved the study (Protocol number 35516720.5.0000.5153). A consent form for participation was sent, where participants could declare their consent or not participate in the research. In the end, the consent form was automatically generated and sent by email to the participants. A pilot study was carried out with about 30 respondents.

To investigate the weight gain, participants recorded their usual weight before the implementation of prophylactic measures of social isolation in Brazil (around March 2020) and their current weight in kilograms. The weight gain was obtained by the difference between the volunteers' current weight and their weight prior to the pandemic. When that difference was positive (>+0.1), volunteers were classified as having weight gain. They also recorded their height in meters. Body mass index (BMI) before and during the pandemic period was calculated and participants were classified as underweight (BMI <18.5 kg/m^2^); normal (BMI: 18.5–24.9 kg/m^2^), overweight (BMI: 25.0–29.9 kg/m^2^), and obese (BMI: >30.0 kg/m^2^) (14). Intention, or otherwise, to change the weight was also collected.

Socioeconomic data (age, gender, home state, per capita income, education level, home residents); labor situation and social isolation occurred by the pandemic; eating behaviors (assessed by Three-Factor Eating Questionnaire) (15); perceived stress (assessed by Perceived Stress Scale) (16); eating habits (meals, food intake, snacking, food delivery, cooking at home, food frequency) (17); and lifestyle (sleep, physical activity, smoke, alcohol, screen time) prior and/or during the COVID-19 pandemic were investigated as predictors of weight gain, according to Liboredo et al. (18).

Data were analyzed using the software Statistical Package for Social Sciences (SPSS Inc., Chicago, IL, USA) version 21.0. Data was demonstrated as median, interquartile interval (Kolmogorov-Smirnov test; *p* < 0.05) or by frequency and absolute numbers. To evaluate the predictors of weight gain, a multivariate logistic regression model was performed. The selection of variables to enter the model was obtained by Spearman correlation (*p* < 0.05). The model was obtained by the backward conditional method. The fit of the models was tested by the Hosmer Lemeshow test (*p* > 0.05). The level of significance adopted was 0.05.

## Results

There were initially 1,496 answers, but 162 answers were excluded, resulting in 1,334 individuals enrolled in this study. Participants were 31 (24–40) years old the majority were women (79.8%) from the Southeast region of Brazil (89.4%).

The median self-reported weight difference (current - before the pandemic) was + 1.34 kg (ranging from −19.0 to + 30.0 kg; interquartile range: −0.7 to + 3.0 kg), 25.0 and 11.5% of volunteers were overweight and obese, respectively before the pandemic, and these percentages increased to 27.7 and 14.0% during quarantine (*p* < 0.001,Mc Necmar test). Weight gain occurred in 58.8% of participants during the period (median 3.0 kg, varying from 0.1 to 30 kg; interquartile interval: 2.0 to 5.0 kg). The self-reported weight and BMI before the pandemic period were similar among individuals who gained and did not gain weight. Most participants who reported weight gain (77.4%; *n* = 681) stated that it was not intentional. However, 25.4% of participants thought they lost weight (median of −2.7 kg, ranging from −19.0 to −0.2 kg; interquartile range: −4.0 to −2.0 kg). The self-reported weight and BMI characteristics are described in Table 1.

**Table 1:** Weight and body mass index characteristics among the volunteers who gained weight or not during the pandemic period.

Characteristics^**^	**Weight gain**		***p*-value**
	**No**	**Yes**	
	**(41.2%; *n* = 549)**	**(58.8%; *n*= 785)**	
Weight before the pandemic period (kg)	64.0 (57.0–77.0)	64.0 (56.0–75.0)	0.275
Weight during the pandemic period (kg)	63.0 (65.0–75.0)	68.0 (60.0–80.0)	<0.001
Weight difference (kg)	−1.5 (−3.0–0.0)	3.0 (2.0–5.0)	<0.001
BMI before the pandemic period (kg/m^2^)	23.6 (21.1–27.2)	23.4 (21.3–26.6)	0.635
BMI during the pandemic period (kg/m^2^)	22.9 (20.6–26.3)	24.7 (22.2–28.2)	<0.001
BMI difference (kg/m^2^)	−0.5 (−1.1–0.0)	1.1 (0.7–1.7)	<0.001
BMI classification before the pandemic period^*^			
Underweight (%)	4.4 (24)	4.8 (38)	
Normal (%)	56.3 (309)	60.6 (476)	0.336
Overweight (%)	27.3 (150)	23.4 (184)	
Obesity (%)	12.0 (66)	11.1 (87)	
BMI classification during pandemic period^*^			
Underweight (%)	6.7 (37)	2.0 (16)	
Normal (%)	60.1 (330)	50.3 (395)	<0.001
Overweight (%)	24.0 (132)	30.2 (237)	
Obesity (%)	9.1 (50)	17.5 (137)	

* *Qui-Square or*

** *Mann-Whitney test; BMI, body mass index*.

There was no association between socioeconomic data and weight gain (*p* > 0.05). However, the perception of time spent in work, social isolation, perceived stress, changes in eating habits, performing the evening snack, uncontrolled and emotional eating during the quarantine were associated with weight gain in univariate analyses (*p* < 0.05) (Table 2). Also, changes in food intake frequency (cereal, bread, fruit, meat, canned products, vegetables, sugary drinks, instant meals, and snacks, candies, and fast food) occurring during pandemic were associated with weight gain in univariate analyses (*p* < 0.05) (Supplementary Figure 1).

**Table 2:** Socioeconomic data, labor situation, social isolation, perceived stress, eating habits and behavior among the volunteers who gained weight or not during the pandemic period in univariate analyses.

**Characteristics**	**Weight gain**		***p*-value**
	**No**	**Yes)**	
	**(41.2%; *n* = 549)**	**(58.8%; *n* = 785)**	
**Socioeconomic**			
Gender^*^(%, ***n*****)**			
Female	40.6 (432)	59.4 (633)	0.403
Male	43.4 (115)	56.6 (150)	
Age^**^(years)	31.0 (24.0–40.0)	31.0 (24.0–39.0)	0.586
Per capita income^**^(U$)	348.6 (154.9–697.1)	325.3 (154.9–697.15)	0.273
Home residentes^*^(%, ***n*****)**			
Living with parents	37.2 (204)	38.3 (301)	0.688
Living with children	23.0 (126)	27.1 (213)	0.085
Education level^*^(%, ***n*****)**			
Graduate or above	41.7 (369)	58.3 (515)	0.596
Undergraduate or below	40.2 (180)	59.8 (268)	
Labor situation^*^(%, ***n*****)**			
Perception of time spent in work (household chores)^*^(%, ***n*****)**			
Increased	60.1 (330)	69.6 (546)	<0.001
The same + reduced	30.4 (219)	39.9 (319)	
Working schedule^*^(%, ***n*****)**			
Full-time work or study at home	38.6 (212)	41.5 (326)	0.307
Full/part-time work or study at home	68.5 (376)	72.1 (566)	0.160
Same situation before pandemic period	11.8 (65)	10.6 (83)	0.480
COVID-19 frontline worker^*^(%, ***n*****)**	5.5(30)	6.5 (51)	0.485
Social isolation^*^(%, ***n*****)**			
Total /Partial	54.1 (297)	60.9% (478)	0.015
Perceived stress^**^(score)	24.0 (19.0–28.0)	22.0 (17.0–27.0)	<0.001
Eating habits^**^			
Number of meals	15.3 (84)	28.7 (225)	<0.001
Increased food intake (%, *n*)	31.7 (174)	77.5 (608)	<0.001
Increased snacking (%, *n*)	30.2 (166)	66.1 (519)	<0.001
Increased food delivery (%, *n*)	44.6 (245)	54.4 (427)	<0.001
Increased homemade meals (%, *n*)	63.9 (351)	69.7 (547)	0.028
Consumption of meals^*^(%, ***n*****)**			
Breakfast	84.7 (465)	83.7 (657)	0.648
Morning snack	33.0 (181)	33.0 (259)	1.000
Lunch	97.1 (533)	97.3 (764)	0.866
Afternoon snack	78.7 (432)	82.3 (646)	0.105
Dinner	79.4 (436)	82.7 (649)	0.135
Evening snack	24.0 (132)	32.4 (254)	0.001
Eating behavior^**^(score)			
Uncontrolled eating	25.9 (11.1–40.7)	37.0 (22.2–51.8)	<0.001
Emotional eating	27.2 (5.5–50.0)	44.4 (16.7–66.7)	<0.001
Cognitive restraint	44.4 (27.8–61.1)	44.4 (27.8–61.1)	0.525

* *Qui-Square or*

** *Mann-Whitney test*.

**Figure 1:**
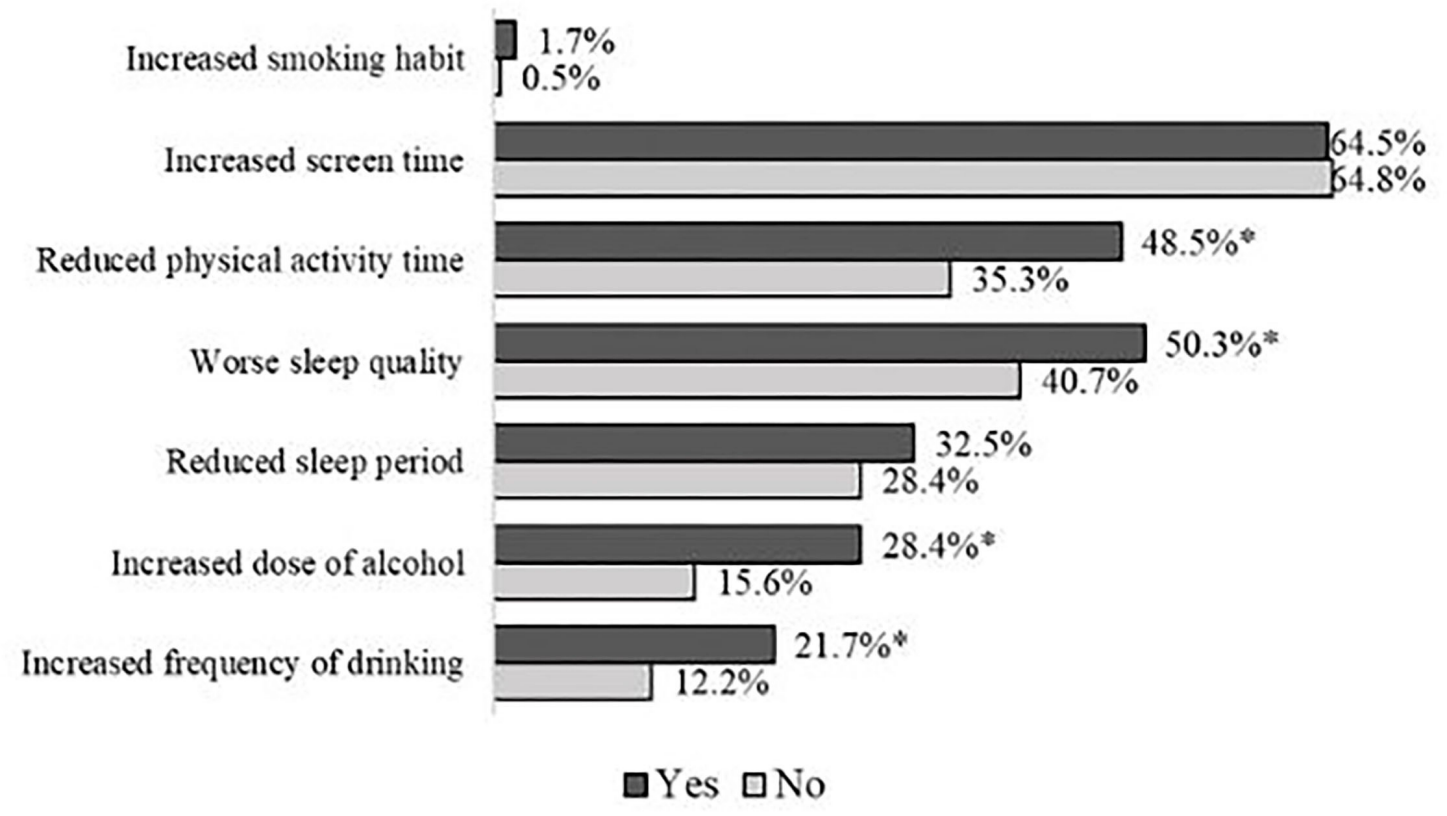
Lifestyle changes among the volunteers who gained weight or not during the pandemic period in univariate analyses. Qui-square test ^*^p < 0.05.

Lifestyle changes during quarantine related to weight gain can be seen in Figure 1. Reduced physical activity time, worse sleep quality, and increased dose and frequency of alcoholic beverages were more frequent in individuals who have weight gain (*p* < 0.05 for all).

Predictors of weight gain during the pandemic period can be seen in Table 3. Increased food intake, snacking, evening snack, time spent at work (including household chores), frequency and dose of alcoholic beverage, fast food, and canned product intake about the period before the pandemic period, and uncontrolled eating increased the chance of weight gain. However, increased physical activity and vegetable intake reduced the chance of weight gain during the quarantine.

**Table 3:** Weight gain predictors among Brazilians during pandemic period in multivariate analyses.

**Predictors of weight gain**	**OR**	**IC 95%**		***p*-value**
**(Prediction of 72.4% of cases; Hosmer Lemeshow = 0.07)**			
**Increased the chance of weight gain**			
Increased food intake	5.405	3.690	7.874	<0.001
Increased snacking	2.860	2.189	3.737	<0.001
Increased frequency of alcoholic beverage intake	1.589	1.087	2.322	0.017
Increased perception of time spent at work (including household chores)	1.581	1.511	2.063	0.001
Eneving snack during the pandemic period	1.538	1.150	2.056	0.004
Increased dose of alcoholic beverage intake (per week)	1.109	1.028	1.195	0.007
Increased fast food intake frequency in relation to the period prior pandemic (times/week)	1.107	1.024	1.197	0.011
Increased frequency incanned products intake in relation to the period prior pandemic (times/week)	1.077	1.005	1.155	0.037
Uncontrolled Eating (score)	1.010	1.004	1.017	0.003
**Reduced the chance of weight gain**			
Physical activity before pandemic period (min/week)	0.997	0.995	0.998	<0.001
Increased in physical activity in relation to the period prior to the pandemic (min)	0.997	0.995	0.998	<0.001
Increased in vegetables intake frequency in relation to the period prior to the pandemic (times/week)	0.923	0.872	0.977	0.006
**Constant**	0.291			0.271

## Discussion

Our study revealed that most survey participants (58.8%) reported weight gain during the COVID-19 quarantine outbreak in Brazil. Other studies reported weight gain in 48.6% among the Italian population (2), 30% in Poland (6), and 22.0% in participants from different nationalities (7). Moreover, in this study, the prevalence of overweight and obesity before and during the pandemic increase significantly. Before the pandemic period, about 2/3 of the Brazilian population was already overweight and obese (11).

These data are worrying since obesity has emerged as an important risk factor for aggravation and mortality for COVID-19 (8). In our study, data were collected 5 months after the decree of social distancing in Brazil. The choice of the period was necessary so that the data collected would reflect changes made after adaptation to the new living conditions. Thus, it is possible to better check the predictors of risk and protection of weight gain during this pandemic scenario.

It is worth mentioning that studies in the literature show that women are more prone to weight gain than men (19), even during the pandemic period (20). Despite this, our study did not observe this scenario since gender was not an independent factor associated with weight gain.

Eating behaviors are relevant influences on food choices and BMI (9). Our findings revealed that increased uncontrolled eating behavior was a predictor of weight gain. Considering the scores, for each point added on the scale of uncontrolled eating, the chance of gaining weight during the pandemic increased by 1%, and this means that the more points, the greater the chance. This behavior is associated with excessive food consumption (21), and it may be a cause and a consequence of being overweight. During the pandemic, uncontrolled eating behavior may be related to the feeling of boredom generated by social isolation, where individuals were forced to stay indoors for an extended period. In this way, overeating may be a way to avoid monotony (2).

Social isolation is related to increased food consumption and the development of obesity (22). In the present study, increased food intake was related to 77.5% of participants who reported weight gain. Additionally, 66.1% of individuals who gained weight indicated increased snacking which is associated with an increase in fat mass and fat percentage (23). These changes in dietary patterns were also observed by Sidor and Rzymski (6) during the COVID-19 lockdown. Unlimited access to food during the quarantine may cause a perturbation of time-restricted feeding (6), which is known to support robust metabolic cycles and has a protective role in dysmetabolism and obesity (24). Sisto et al. (25) also demonstrated a relationship between symptoms of anxiety and depression with the consumption of snacks, increased hunger, and impulsive eating, since suffering negatively impacts dietary treatments (25). The state of being depressed or the worsening of this condition are precursors for weight gain and recovery (26). On the other hand, Barcin-Güzeldere and Devrim-Lanpir (27) suggested that the stress transmitted by the COVID-19 pandemic can cause a reduction in appetite since some will want to increase the consumption of certain foods as a way to ward off emotions. Others do the opposite, that is, reducing their consumption.

The COVID-19 pandemic has brought challenges, as it unequally affects mental health in women and men since women seem to be more impacted by the quarantine, and depression, anxiety, and stress can intensify. There is a dangerous association between COVID-19 infection, depression, and cardiovascular disease (28). It is also worth noting how cardiovascular diseases are associated with deaths from COVID-19. Social distance can minimize disease infestation; however, the restrictions imposed by pandemics may directly influence lifestyle (29).

The association between perceived stress and detected weight gain may be related to the predominant gender of the sample, composed of about 80% females. Torres and Nowson (30) showed the relationship between impulsive eating when facing a stressful situation and obesity only in females. The authors also explained that women tend to use food as a means of dealing with stress, while men seek to consume alcohol or tobacco (30).

One eating occasion also had a significant result in this study: evening snack was a predictor for weight gain during the COVID-19 pandemic. This result agrees with previous research that showed an association between a higher frequency of snacking at night and an increased risk of developing obesity, especially in overweight individuals (31).

Furthermore, unhealthy eating habits may contribute to weight gain. In the multiple logistic regression analyses, increased intake of fast food and canned products were associated with a greater chance of weight gain (10.7 and 7.7%, respectively), while the increase in the frequency of vegetable consumption during the pandemic period reduced the likelihood of weight gain by 7.7%. Likewise, worsening of the eating pattern was observed in studies carried out with other populations during the COVID-19 pandemic (32). The association between fast food and canned products consumption and weight gain has been observed in previous studies (33). Fast food products are high in fat, sugars, and energy density, and they are often served in large portion sizes; also, canned products are among the ultra-processed foods that provide more calories (34). An increase in the consumption of these foods during the COVID-19 pandemic may be related to a tendency to experience at home some socialization habits that are traditionally external, such as meeting with friends, enjoying an aperitif, and eating out (35). On the other hand, foods of plant origin have numerous nutrients in a low amount of calories, which make them ideal for preventing overweight and obesity (34).

Regarding lifestyle habits, alcohol intake increased the chance for weight gain during quarantine time, and extended stays at home could also directly affect alcohol consumption. The increase in the frequency (58.9% greater chance) and the dose of alcohol (10.9% greater chance) intake affected the chance of weight gain in the present study. Alcohol is the most consumed substance by different populations, and its excessive consumption is related to weight gain among individuals (36). This possible association can be explained by variation on the frequency and amount consumed, if there is excessive consumption of calories, whether it exceeds the daily caloric needs of individuals. These factors can possibly lead to weight gain (36). The increase in the frequency of consumption of alcohol may be related to the attempt to combat stress and other negative emotions caused by social isolation (6).

On the other hand, physical activity before quarantine and the increase in practice during the pandemic reduced the chance for increasing body weight in the present study. This leads us to consider that maintaining an active lifestyle during a period of social isolation is crucial (37). In addition, physical exercise improves immune function, increasing the response to virus infections, such as SARS-CoV-2 (38). This is a fascinating result since most participants are female, and according to the literature, women tend to be less active than men (28).

Increased perception of work time during the pandemic also raised the chance of weight gain by 58.1%. As a consequence of social isolation, changes in the work modality of a large part of the population were necessary, with the “smart” work mode being implemented (39). As a result of this adaptation, it was observed that the working time increased (40). With the expanded demand for working at home, individuals are remaining sat for more hours daily, consequently increasing sedentary behavior, as has been seen in other populations during quarantine for COVID−19 (41).

Although this study provides insight into how epidemic-related social isolation can affect weight gain, some limitations also need to be considered. First, anthropometric data was not measured directly but self-reported. However, this approach is commonly used in several studies during the pandemic period (2, 6). Also, the sample may not be representative because most responses were female participants, and hence the results may be more linked to this audience.

The main strengths of this study were that it was carried out with the Brazilian population and that the data were collected 5 months after the ruling of social isolation in Brazil, which made it possible to assess changes in weight after a period of adaptation to a relatively rigorous quarantine in the country. Furthermore, our study provides important results that may be used to establish strategies for weight control in possible situations of future social isolation. Thus, this study allows for more comprehensive data on predictors of weight gain in such population.

The quarantine period affected the population's lifestyle and eating habits, and these, in turn, caused a direct impact on their weight gain in Brazil. Most participants reported weight gain during the pandemic. Food intake, snacking, time spent at work, including household chores, and intake of fast food and canned products increased compared to the period before the pandemic. Evening snacks, higher frequency and dose of alcoholic beverage intake, and uncontrolled eating increased the chance of weight gain. On the other hand, physical activity before the pandemic period as well as increased physical activity and vegetable intake during the quarantine reduced the chance for increasing body weight.

## Data Availability Statement

The original contributions presented in the study are included in the article/Supplementary Material, further inquiries can be directed to the corresponding author/s.

## Ethics Statement

The studies involving human participants were reviewed and approved by Ethics Committee of the Federal University of Vicosa, Minas Gerais, Brazil. The patients/participants provided their written informed consent to participate in this study.

## Author Contributions

LA, LF, JL, and CD: equally contributed to the conception and design of the research. LA, LF, JL, CD, and LO: contributed to the acquisition of the data. LA and LF: contributed to the statistical analysis. MD, JL, TS, LA, and LF: contributed to the interpretation of the data. MD and JL: drafted the manuscript. All authors critically revised the manuscript, agree to be fully accountable for ensuring the integrity and accuracy of the work, and read and approved the final manuscript.

## Conflict of Interest

The authors declare that the research was conducted in the absence of any commercial or financial relationships that could be construed as a potential conflict of interest.

## Publisher's Note

All claims expressed in this article are solely those of the authors and do not necessarily represent those of their affiliated organizations, or those of the publisher, the editors and the reviewers. Any product that may be evaluated in this article, or claim that may be made by its manufacturer, is not guaranteed or endorsed by the publisher.

## References

[1] Brasil [Internet]. Coronavírus Brasil. 2021 [cited 2021 Jul 20]. Available online at: https://covid.saude.gov.br/ (accessed November 18, 2021).

[2] Di Renzo L Gualtieri P Pivari F Soldati L Attinà A. Eating habits and lifestyle changes during COVID-19 lockdown: an Italian survey. J Trans Med. (2020) 18:229. 10.1186/s12967-020-02399-5 32513197 PMC7278251

[3] Reyes-Olavarría D Latorre-Román PÁ Guzmán-Guzmán IP Jerez-Mayorga D Caamaño-Navarrete F Delgado-Floody P. Positive and negative changes in food habits, physical activity patterns, and weight status during COVID-19 confinement: associated factors in the chilean population. Int J Environ Res Public Health. (2020) 17:5431. 10.3390/ijerph17155431 32731509 PMC7432624

[4] Lim S Shin S Nam GE Jung CH Koo BK. Proper management of people with obesity during the COVID-19 Pandemic. J Obes Metab Syndr. (2020) 29:84–98. 10.7570/jomes20056 32544885 PMC7338495

[5] Cherikh F Frey S Bel C Attanasi G Alifano M Iannelli A. Behavioral food addiction during lockdown: time for awareness, time to prepare the aftermath. Obes Surg. (2020) 13:1–3. 10.1007/s11695-020-04649-3 32405909 PMC7220644

[6] Sidor A Rzymski P. Dietary choices and habits during COVID-19 lockdown: experience from Poland. Nutrients. (2020) 12:1657. 10.3390/nu12061657 32503173 PMC7352682

[7] Zachary Z Brianna F Brianna L Garrett P Jade W. Self-quarantine and weight gain related risk factors during the COVID-19 pandemic. Obes Res Clin Pract. (2020) 14: 210–16. 10.1016/j.orcp.2020.05.004 32460966 PMC7241331

[8] Petrilli CM Jones SA Yang J Rajagopalan H O'Donnell L. Factors associated with hospital admission and critical illness among 5,279 people with coronavirus disease 2019 in New York City: prospective cohort study. BMJ (Clin Res ed). (2020) 369:m1966. 10.1136/bmj.m1966 32444366 PMC7243801

[9] Almandoz JP Xie L Schellinger JN Mathew MS Gazda C. Impact of COVID-19 stay-at-home orders on weight-related behaviours among patients with obesity. Clin Obes. (2020) 10:e12386. 10.1111/cob.12386 32515555 PMC7300461

[10] Lavie CJ Sanchis-Gomar F Henry BM Lippi G. COVID-19 and obesity: links and risks. Expert review of endocrinology & metabolism. (2020) 15: 15–216. 10.1080/17446651.2020.1767589 32441223

[11] Brasil Ministério da Saúde IBGE 2019. Pesquisa nacional de saúde: 2019 : atenção primária à saúde e informações antropométricas : Brasil / IBGE, Coordenação de Trabalho e Rendimento [cited 2020]. Available online at: https://biblioteca.ibge.gov.br/index.php/biblioteca-catalogo?view=detalhes&id=2101758 (accessed December 20, 2020).

[12] Verticchio DFR Verticchio NM. Os impactos do isolamento social sobre as mudanças no comportamento alimentar e ganho de peso durante a pandemia do COVID-19 em Belo Horizonte e região metropolitana, Estado de Minas Gerais, Brasil. Res Soc Develop. (2020) 9:e460997206. 10.33448/rsd-v9i9.7206

[13] Botero JP Frah BQ Correia MA Lofrano-Prado MC Cucato GG. Impacto da permanência em casa e do isolamento social, em função da COVID-19, sobre o nível de atividade física e o comportamento sedentário em adultos brasileiros. Einstein (São Paulo). (2020) 19:1–6. 10.31744/einstein_journal/2021AE6156 33681886 PMC7909004

[14] WHO Expert Committee on Physical Status : The Use and Interpretation of Anthropometry (1993). Geneva, S. & Organization, W.H., 1995, Physical status : the use of and interpretation of anthropometry, report of a WHO expert committee, World Health Organization. 8594834

[15] Natacci LC Ferreira Júnior M. The three factor eating questionnaire - R21: tradução para o português e aplicação em mulheres brasileiras. Revista de Nutrição. (2011) 24:383–94. 10.1590/S1415-52732011000300002

[16] Reis RS Hino AAF Añez CRR. Perceived stress scale: reliability and validity study in Brazil. J Health Psychol. (2010) 15:107–14. 10.1177/1359105309346343 20064889

[17] Brasil. SISVAN [Internet]. 2017. Food Consumption Markers. Available online at: http://sisaps.saude.gov.br/sisvan/public/file/ficha_marcadores_alimentar.pdf (accessed August 10, 2020).

[18] Liboredo JC Anastácio LR Ferreira LG Oliveira LA Della Lucia CM. Quarantine during COVID-19 Outbreak: eating behavior, perceived stress, and their independently associated factors in a Brazilian sample. Frontiers in Nutrition. (2021) 8:704619. 10.3389/fnut.2021.704619 34381806 PMC8349978

[19] Kanter R Caballero B. Global gender disparities in obesity: a review. Am J Clin Nutr. (2012) 3:491–8. 10.3945/an.112.002063 22797984 PMC3649717

[20] Mulugeta W Desalegn H Solomon S. Impact of the COVID-19 pandemic lockdown on weight status and factors associated with weight gain among adults in Massachusetts. Clin Obes. (2021) 11:1–8. 10.1111/cob.12453 33855789 PMC8250379

[21] Vainik U García-García I Dagher A. Uncontrolled eating: a unifying heritable trait linked with obesity, overeating, personality and the brain. Eur J Neurosci. (2019) 50:2430–45. 10.1111/ejn.14352 30667547

[22] Nonogaki K Nozue K Oka Y. Social isolation affects the development of obesity and type 2 diabetes in mice. Endocrinol. (2007) 148:4658–66. 10.1210/en.2007-0296 17640995

[23] Larsen SC Heitmann BL. More frequent intake of regular meals and less frequent snacking are weakly associated with lower long-term gains in body mass index and fat mass in middle-aged Men and Women. J Nutr. (2019) 149:824–30. 10.1093/jn/nxy326 31034009

[24] Zarrinpar A Chaix A Panda S. Daily eating patterns and their impact on health and disease. Trends Endocrinol Metab. (2016) 27:69–83. 10.1016/j.tem.2015.11.007 26706567 PMC5081399

[25] Sisto A Vicinanza F Tuccinardi D Watanabe M Gallo IF D'Alessio R. The psychological impact of COVID-19 pandemic on patients included in a bariatric surgery program. Eat Weight Disord. (2021) 26:1737–47. 10.1007/s40519-020-00988-3 32857287 PMC7453189

[26] Odom J Zalesin KC Washington TL Miller WW Hakmeh B Zaremba DL. Behavioral predictors of weight regain after bariatric surgery. Obesity Sugery. (2010) 20:349–56. 10.1007/s11695-009-9895-6 19554382

[27] Barcin-Güzeldere HK Devrim-Lanpir A. The association between body mass index, emotional eating and perceived stress during COVID-19 partial quarantine in healthy adults. Nutr Soc. (2021) 21: 1–23. 10.1017/S1368980021002974 34261563 PMC8365042

[28] Bucciarelli V Nasi M Bianco F Sferovic J Ivkovic V Gallina S. Depression pandemic and cardiovascular risk in the COVID-19 era and long COVID syndrome: gender makes a difference. Trends Cardiovasc Med. (2022) 32:12–7. 10.1016/j.tcm.2021.09.009 34619336 PMC8490128

[29] Mattioli AV Nasi M Cocchi C Farinetti A. COVID 19 outbreak: impact of the quarantine-induced stress on cardiovascular disease risk burden. Future Cardiol. (2020) 4: 1–4. 10.2217/fca-2020-0055 32351128 PMC7202322

[30] Torres SJ Nowson CA. Relationship between stress, eating behavior, and obesity. Nutrition. (2007) 23:887–94. 10.1016/j.nut.2007.08.008 17869482

[31] Barrington WE. Beresford SAA. Eating Occasions, Obesity and Related Behaviors in Working Adults: Does it Matter When You Snack?. Nutrients. (2019) 11:2320. 10.3390/nu11102320 31581416 PMC6835708

[32] Eftimov T Popovski G Petković M Seljak BK Kocev D. COVID-19 pandemic changes the food consumption patterns. Trends Food Sci Technol. (2020) 104:268–72. 10.1016/j.tifs.2020.08.017 32905099 PMC7462788

[33] Pereira MA Kartashov AI Ebbeling CB Van Horn L Slattery ML. Fast-food habits, weight gain, and insulin resistance (the CARDIA study): 15-year prospective analysis. Lancet. (2005) 365:36–42. 10.1016/S0140-6736(04)17663-0 15639678

[34] Brasil [Internet]. 2014. Guia alimentar para a população brasileira. 158. Available online at: https://bvsms.saude.gov.br/bvs/publicacoes/guia_alimentar_populacao_brasileira_2ed.pdf (accessed December 10, 2020).

[35] Bracale R Vaccaro CM. Changes in food choice following restrictive measures due to Covid-19. Nutr Metab Cardiovas Dis. (2020) 30:1423–6. 10.1016/j.numecd.2020.05.027 32600957 PMC7832660

[36] López-Suárez A. Burden of cancer attributable to obesity, type 2 diabetes and associated risk factors. Metab. (2019) 92:136–46. 10.1016/j.metabol.2018.10.013 30412695

[37] Martinez-Ferran M Guía-Galipienso F de. la, Sanchis-Gomar F, Pareja-Galeano H. metabolic impacts of confinement during the Covid-19 pandemic due to modified diet and physical activity habits. Nutrients. (2020) 12:1549. 10.3390/nu12061549 32466598 PMC7352228

[38] Górnicka M Drywień ME Zielinska MA Hamułka J. Dietary and lifestyle changes during Covid-19 and the subsequent lockdowns among polish adults: a cross-sectional online survey PLifeCOVID-19 study. Nutrients. (2020) 12:2324. 10.3390/nu12082324 32756458 PMC7468840

[39] Barrea L Pugliese G Framondi L Di Matteo R Lausisio D. Does Sars-Cov-2 threaten our dreams? Effect of quarantine on sleep quality and body mass index. J Transl Med. (2020) 18:318. 10.1186/s12967-020-02465-y 32811530 PMC7432549

[40] Coutinho M P L Costa FG Sá JGC Coutinho ML. Quarentena e aulas remotas representações sociais de universitários da saúde. Diálogos em Saúde. (2020) 3:119–30.

[41] Ammar A Brach M Trabelsi K Chtourou H Boukhris O. E?ects of COVID-19 Home confinement on eating behavior and physical activity: results of the ECLB-COVID19 international online survey. Nutrients. (2020) 12:1583. 10.3390/nu12061583 32481594 PMC7352706

