# Supplementary figures and images for: Incidence and Associated Factors of Weight Gain During the Covid-19 Pandemic
Source: Front Nutr. 2022 Feb 24;9:818632. doi: 10.3389/fnut.2022.818632 (PMC8908378; doi:10.3389/fnut.2022.818632)

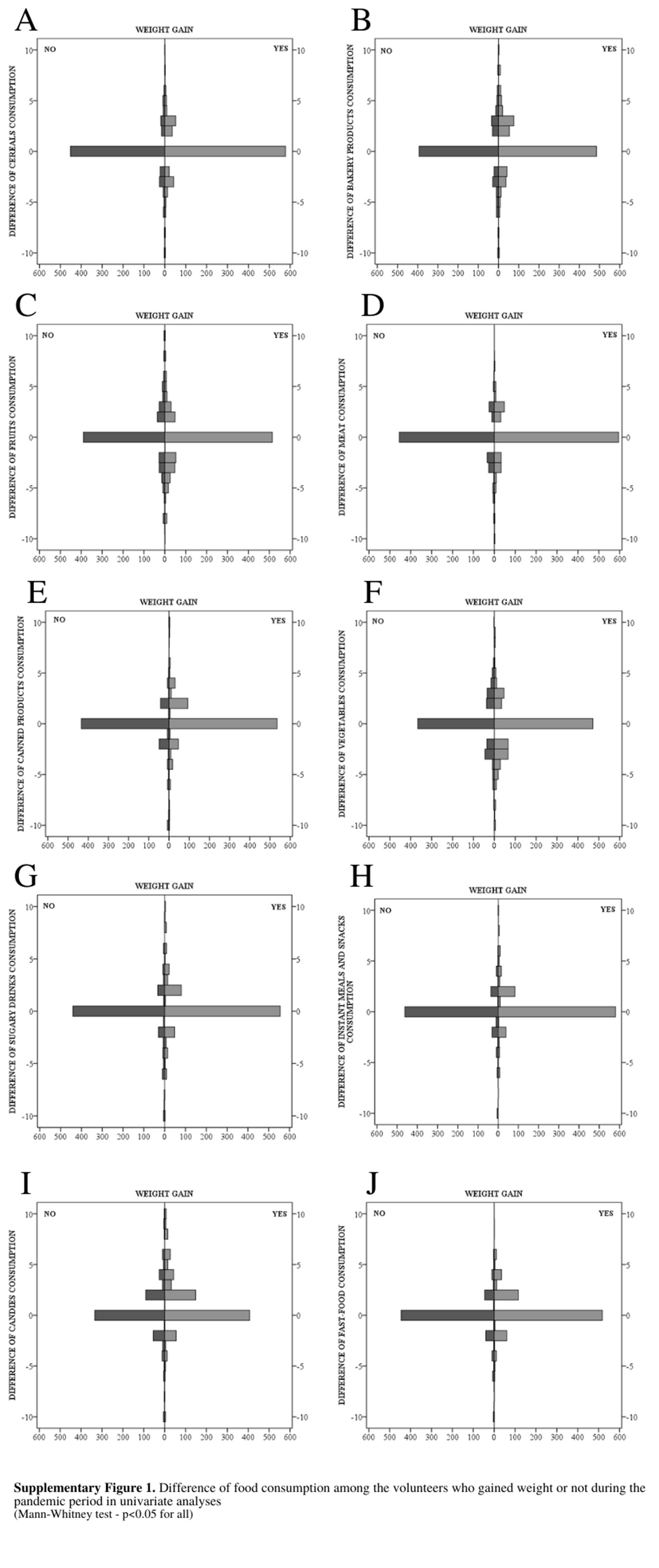

Supplement: Supplementary file 1 [file Image_1.PNG]
